# Editorial: Women in phage biology: 2022

**DOI:** 10.3389/fmicb.2023.1193890

**Published:** 2023-04-24

**Authors:** Alicja Wegrzyn

**Affiliations:** University Center for Applied and Interdisciplinary Research, University of Gdansk, Gdansk, Poland

**Keywords:** bacteriophages, virus-host interactions, genomics, genetic recombination, phage therapy

Bacteriophages (or shortly “phages”) are viruses infecting prokaryotic organisms. They are considered the most abundant biological entities in the world, as the number of virions occurring on Earth is estimated to be around 10^31^. These viruses are found in all known habitats, wherever living prokaryotic cells can be found (Dion et al., 2020). In fact, biodiversity of phages is enormous, not only when thinking globally, but also if a single environment is considered (Jurczak-Kurek et al., 2016; Olszak et al., 2017). Because bacteriophages infect bacterial cells and propagate in them, they effectively control number of these prokaryotic organisms. This indicates a crucial role played by these viruses in the environment, as without their action, bacterial populations would grow in almost uncontrolled manner, disturbing the biological balance in most of habitats. On the other hand, bacteriophages are also used by humans in many fields. They are widely employed in various areas of genetic engineering and biotechnology (Elois et al., 2023). The phage display technology can be indicated as one of many examples of the employment of bacteriophage-based methods used in various practical applications, including development of novel materials and sophisticated diagnostic and therapeutic approaches in medicine and veterinary medicine (Jaroszewicz et al., 2022; Grabowski et al., 2023). In fact, the use of bacteriophages to combat bacterial infections, called the phage therapy, is a quickly expanding field which provides a hope for development of novel and effective anti-bacterial treatments. Those are especially desired in the era of the antibiotic crisis, caused by appearance of multidrug-resistant strains of pathogenic bacteria (Baral, 2023; Strathdee et al., 2023).

In the light of the above mentioned importance of bacteriophages in the environment and human civilization, it is clear that extensive studies on these viruses are mandatory. This statement is substantiated not only because our knowledge in the field of phage biodiversity is still scarce, but also because development of novel phage-based applications require discovery of as yet unknown bacteriophages with desired properties, like effective infection of specific bacterial hosts. Moreover, improvement of already existing and introduction of novel methods in molecular biology and genetics requires more detailed understanding of processes and regulatory circuits operating during the bacteriophage infection. Therefore, one the special Research Topics of *Frontiers in Microbiology* has been focused on studies on bacteriophages. Especially, this collection of articles aimed at celebrating the achievements of women in this field. Unfortunately, there is still gender disparity in science, technology, engineering, and mathematics, as according to UNESCO Institute for Statistics, only 30% of the world's researchers are women. On the other hand, science and gender equality are essential to ensure sustainable development, as indicated by UNECSO. Thus, this Research Topic acted as a platform to promote the work of female researchers and highlight the diversity of research performed across the entire breadth of phage biology. The current collection consists of four articles, well representing both the diversity of studies on phages and excellent contribution of women as senior researchers, leading the projects and supervising the studies.

As reported previously and indicated in the preceding paragraphs, isolation and characterization of previously unknown phages infecting pathogenic bacteria is crucial for developing effective phage therapy (Baral, 2023; Strathdee et al., 2023). This is especially important as there is a huge diversity of pathogenic bacterial strains, and bacteria can develop resistance to single phages. The work by Zhang et al. described the identification and characterization of a newly discovered bacteriophage vB_StaM_SA1 which infects *Staphylococcus aureus*. Interestingly, this phage reveals a relatively broad host range, being able to propagate in cells of many strains of this bacterial species. This is possible perhaps due to the presence of the large genome of this virus, consisting of over 260 kb, which encodes multiple RNA polymerase subunits and many other proteins potentially allowing the phage to use cellular machineries present in multiple *S. aureus* hosts. Due to this large genome, vB_StaM_SA1 can be classified as a jumbo bacteriophage. Interestingly, this phage encodes also at least two lysins, active in destroying bacterial cell envelopes and killing the cells. Therefore, both the phage vB_StaM_SA1 itself and lysins encoded in its genome can be considered as promising tools for a novel antibacterial therapy.

Another phage with a relatively broad host range has been described by Mondal et al.. This newly isolated phage, named STWB21, infects several serovars of *Salmonella enterica*, including *S. Typhi, S. Paratyphi, S. Typhimurium*, and *S. Enteritidis*. Apart from this feature, some other properties of STWB21 make this bacteriophage especially promising in its use for the phage therapy. Among them, there are high stabilities in a relatively broad range of pH values (between 4 and 11) and temperatures (between 4 and 50°C), lytic mode of development (supported by a lack of genes responsible for lysogenization), and a short latent period (25 min). The authors suggested that apart from phage therapy, STWB21 might be potentially used in biocontrol approaches and in the food industry to eliminate *S. enterica*.

Quite different approach was presented in the work by Stoos et al. who aimed to develop a bacteriophage-based method for monitoring the presence of *Vibrio parahaemolyticus* which contaminates shellfish and can cause foodborne outbreaks. By testing samples of coastal waters, they have identified four bacteriophages that are able to infect different sets of *V. parahaemolyticus* isolates. Therefore, this study provides a basis for construction of a specific bio-test, useful in detection of pathogenic *V. parahaemolyticus* strains.

The fourth article in this collection, published by Bednarek et al., was focused on developing a new method for functional analyses of already known phage genomes rather than on characterization of newly isolated bacteriophages. Such an analytical tool is especially desirable as even in model phages, the functions of many genes remain unknown. The authors have described a novel procedure allowing an efficient traceless modification of the genome of bacteriophage P1. This new method is based on homologous recombination with enrichment in double recombinants. Importantly, it can be useful in introducing targeted mutations in the genomes of various phages which should facilitate functional annotations of genes of these viruses.

In summary, this collection of articles contain papers describing isolation and characterization of previously unknown bacteriophages which may be useful in developing phage therapy and efficient monitoring of the presence of pathogenic bacteria, as well as development of a novel, useful method for functional analyses of phage genes. Therefore, researchers are encouraged to read these articles which contribute significantly to expand our knowledge on bacteriophages and their potential applications.

## Author contributions

The author confirms being the sole contributor of this work and has approved it for publication.

## References

[B1] Baral B. (2023). Phages against killer superbugs: An enticing strategy against antibiotics-resistant pathogens. Front. Pharmacol. 14, 1036051. 10.3389/fphar.2023.1036051 36762109 PMC9902939

[B2] Dion M. B. Oechslin F. Moineau S. (2020). Phage diversity, genomics and phylogeny. Nat. Rev. Microbiol. 18, 125–138. 10.1038/s41579-019-0311-5 32015529

[B3] Elois M. A. Silva R. D. Pilati G. V. T. Rodríguez-Lázaro D. Fongaro G. (2023). Bacteriophages as biotechnological tools. Viruses 15, 349. 10.3390/v15020349 36851563 PMC9963553

[B4] Grabowski Ł. Pierzynowska K. Gaffke L. Cyske Z. Mincewicz G. Wegrzyn G. (2023). The use of phage display systems to combat infectious diseases in poultry: diagnostic, vaccine, and therapeutic approaches. J. Appl. Microbiol. 134, lxac012. 10.1093/jambio/lxac012 36626750

[B5] Jaroszewicz W. Morcinek-Orłowska J. Pierzynowska K. Gaffke L. Wegrzyn G. (2022). Phage display and other peptide display technologies. FEMS Microbiol. Rev. 46, fuab052. 10.1093/femsre/fuab052 34673942

[B6] Jurczak-Kurek A. Gasior T. Nejman-Faleńczyk B. Bloch S. Dydecka A. Topka G.. (2016). Biodiversity of bacteriophages: morphological and biological properties of a large group of phages isolated from urban sewage. Sci. Rep. 6, 34338. 10.1038/srep34338 27698408 PMC5048108

[B7] Olszak T. Latka A. Roszniowski B. Valvano M. A. Drulis-Kawa Z. (2017). Phage life cycles behind bacterial biodiversity. Curr. Med. Chem. 24, 3987–4001. 10.2174/0929867324666170413100136 28412903

[B8] Strathdee S. A. Hatfull G. F. Mutalik V. K. Schooley R. T. (2023). Phage therapy: From biological mechanisms to future directions. Cell 186, 17–31. 10.1016/j.cell.2022.11.017 36608652 PMC9827498

